# Study of the Biochemical Biodiversity of Camel Milk

**DOI:** 10.1155/2019/2517293

**Published:** 2019-04-09

**Authors:** Sara Bouhaddaoui, Rachida Chabir, Faouzi Errachidi, Lahsen El Ghadraoui, Bouchra El Khalfi, Meryem Benjelloun, Abdelaziz Soukri

**Affiliations:** ^1^Laboratory of Physiology, Molecular Genetics and Biotechnology, Hassan II University, BP 8110, Casablanca, Morocco; ^2^Laboratory of Human Pathology, Biomedicine and Environment, Sidi Mohammed Ben Abdellah University, BP 30050 Fez, Morocco; ^3^Laboratory of Functional Ecology and Environment, Sidi Mohammed Ben Abdellah University, BP 30050 Fez, Morocco

## Abstract

Due to its balanced composition in basic nutrients, its richness in vitamins and in minerals, camel milk has a special place in the daily diet of southern populations. The present study aimed at examining some physicochemical and biochemical characteristics of camel milk (*Camelus dromedarius*) in Morocco. To achieve this aim, three batches of samples of camel milk were collected from various regions (Dakhla, Errachida, and Fès-Meknes) undergoing physicochemical and biochemical analyses. Results showed that Moroccan camel milk is characterized by slight hydronium potential (pH=6.5), low Dornic acidity (15°D), low density (1.029 kg/l), and high content of ashes (8.06 g/l). Likewise, samples had a high content of macronutrients (Fats: 34.09 g/l; Proteins: 32.4 g/l; Sugar: 49.8 g/l) and micronutrients (Vitamin C: 27.53 mg/l; Flavonoids: 29.05 mg EQ/l: total phenolic compounds: 35.45mg GAE/l). In this respect, working on multiple specimens from different Moroccan regions highlighted an analytical diversity from the south to the north. Comparative study of samples from numerous territories all over the world has confirmed this diversity. North African milk is characterized by high content of proteins, fats, and sugar. On the other hand, oriental milk is peculiarized by high vitamin C content.

## 1. Introduction

Dromedary (*Camelus dromedarius*) is a species that exists in several desert regions of the world [1]. In Morocco, it is located especially in the southern Saharan area. Besides it is known for its resistance to drastic conditions prevailing in these zones [2]. Further, it plays an important socioeconomic role in the preservation of their optimal-potential life [3, 4]. What is more, dromedary's productions are variable: it can be valued for its milk, meat, wool, and leather. The benefits of dromedary milk can be correlated to their composition, mainly to the high concentration of potassium, magnesium, albumin (protein), and vitamins [5]. Camel milk can be considered as a food of high nutritive and therapeutic applications [6]. Indeed, camel milk has recently been recognized for several therapeutic properties such as being anticancer [7] and antidiabetic [8]. It has also been recommended for children allergic to bovine milk [9]. Currently, nutrition labels on food and dairy products indicate levels not only of protein, fat, carbohydrates, sodium, calcium, and vitamins but also of some special ingredients as saturated, unsaturated, omega-3, conjugated, and transfatty acids. This allows us to consider that some dairy products may be superior to others and ask which animal feeding system, such as pasturing versus barn feeding, is good, or which animal species produces a more suitable and preferable human food. Moreover some children and sick patients need to know which milk is closest to human milk [10]. A number of chronic diseases resulting from oxidative stress uncontrolled by the excess of free radicals and other reactive oxygen are clearly presented in human body [11]. To handle this problem, scientific research proved the potential therapeutic properties of milk by demonstrating the strong antioxidant activity of this biological fluid [12]. Morocco is one of the producing countries of camel's milk, which has seen this emerging awareness regarding the valorization of this animal heritage to diversify the range of dairy products. In this perspective, ministerial and academicals institutions have been looking into upgrading this sector. In this context, in Morocco, few studies were previously published on camel's milk composition. In this respect, more studies are needed for a better characterization of this product, to establish its national quality standards and specifies. Consequently, this might encourage the blossoming camel's sector in the south of Morocco. So, the aim of the present study is to assess nutritional diversity of Moroccan dromedary milk and its comparison with the ones of North Africa and the East.

## 2. Material and Methods

### 2.1. Biological Material

**Figure 1:**
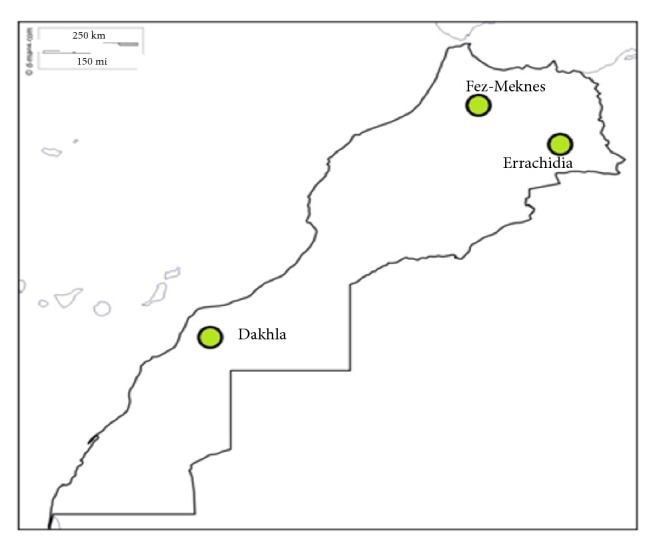
Geographic distribution of the three studied samples of Camel's milk.

Three groups of camel milk samples have been collected from various regions in Morocco: Dakhla in the south, Fez and Meknes in the center, and Errachidia in the east Figure 1. For this reason specimens have been transported to the laboratory in sterile bottles to undergo both physicochemical and biochemical analysis, including measurement of pH, density, titratable acidity, ashes, fats, proteins, sugar, and phenolic compounds (total polyphenols and flavonoids). As a template we have used goat's and cow's milk.

The regions where the milk samples have been collected from are marked by a variety of climates. First Fes-Meknes is subject to a semiarid climate, where average precipitation does not exceed 250 mm per year. The winter is very cold and snowy, with almost daily frosts and an insignificant number of days without thaw. Second Errachidia is characterized by a desert climate. Indeed, its climate is marked by large variations of temperatures (hot summer, cold winter). The winds that usually blow in the spring make the temperatures extremely high. Finally Dakhla is located on the Atlantic coast in the south of Morocco, on the shores of the Dakhla Gulf. On the other side, Dakhla has a desert climate but tempered by the cold Canary ocean current along the North Atlantic coastline, as well. In this case the average of maximum temperature range is from 22°C to 27°C. Yet, annual average precipitation is of 33 mm and is distributed on average 17 days per year. Thus the climate of the coast, which is cooler, contrasts with that of the interior of the country, where, in summer, the temperature can well exceed 48°C.

### 2.2. Physicochemical and Biochemical Characteristics of Camel's Milk

The pH value has been determined at room temperature (20°C) by pH-meter (inolab). The titratable acidity, expressed in Dornic Degree (°D), was determined according to standardized methods by titration with NaOH (N=9) in the presence of phenolphthalein [21]. Besides density has been determined according to AOAC [22]. As regards ashes determination, it was carried out by incinerating the milk at a temperature of 530°C ± 20°C for 4 hours. In this case the result obtained matches with the content of milk ashes, expressed in g/l [22]. In addition fats contents are determined by (Bligh and Dyer, 1959) [23]. Moreover proteins content has been determined according to (Lowry et al., 1951) [24]. Reducing sugar content has been defined by the method of Mailler, 2014 [25]. Furthermore, vitamin C content expressed in (mg/l) was set according to standardized titration methods for iodine (N=0.1) in the presence of starch [14]. Also, total phenolic compounds have been tested by (Singleton and Rossi, 1965) [26]. However, flavonoid content has been established by the aluminum trichloride method (AlCl3) according to (Bahorun et al., 1996) [27].

### 2.3. Statistical Analysis

Outcomes are expressed by mean of three replications ± standard deviation. Statistical analysis has been done by ANOVA one-way using Excel 2007 software. P ≤ 0.05 has been considered to be statistically significant.

## 3. Results and Discussion

### 3.1. The Effect of Geographical Distribution on Physicochemical and Biochemical Characterization of Camel's Milk

Based on the results shown in Table 1, we have noticed that milk samples from various regions have different physicochemical characteristics. In this respect the pH (6.43) and the density (1.028 kg / l) of milk collected from Dakhla are lower when compared to others from (Fès-Meknes, Errachidia). On the other side, milk pertaining to Errachidia is less acidic than the one of both Dakhla and Fès-Meknes; it contains high lactose content. Ashes content of the milk withdrawn in Errachidia is in the order of 8,3 g/l and, therefore, appears to be high in comparison with the milk from both Dakhla and Fez-Meknes regions. Moreover the percentage of fats displays that milk from Dakhla appears to be richer in fats (39 g/l) than the one from the other two regions (Fes-Meknes and Errachidia). Further milk from Fes-Meknes has the highest proteins content (33 g/l). Moreover, camel milk from the three regions is characterized by a high content of vitamin C.

The outcomes of physicochemical and biochemical comparative study showed that the average pH value of the collected camel's milk is equal to 6.5, and it is in the order of 6.7 for goat's milk while cow's milk has a pH of 6.79. This result has been reported by some previous studies in different parts of the world [14, 16, 28] (pH = 6.40). In this respect the diversity of camel milk pH values may be due to stage of lactation and type of food. In this work, we have thought that the variation is due to climatic conditions. Besides, it may also vary depending on the rate of vitamin C (ascorbic acid) which is higher in camel milk.

The analyzed camel's milk samples have a titratable acidity in the range of 15°D. This value is lower when it is compared to the one of cow's (18°D) and goat's milk (17°D). These results are similar to those of Abu-lehia (1994) [29] in Saudi Arabia (15°D) and Sboui (2009) and Kamoun (1994) 15.6°D and 17.2°D, respectively, in Tunisia [16, 30]. Besides, the density of camel's milk is in the order of 1.029 kg/l, which is slightly lower than cow's milk and goat's milk density. Besides, this value is close to that made by Siboukeur (2007) [14] (d =1.023 kg/l) and Sboui (2009) [16] (d = 1.020 kg/l). These authors have reported that density depends on the dry matter content, fats content, temperature, and animal diet. In these cases the fats content achieved is equal to 35.6 g/l. Moreover it appears to be higher when it is compared to that of cow's milk which is in the order of 36 g/l and lower than that of goat's milk 41 g/l. In addition, templates acquired are in the range of work stated by (Boudjnah, 2012) [31] 30 g/l. Indeed, changes in percentage of fats may be related to cattle feed and lactation.

Results have demonstrated that camel's milk contains 32.6 g/l proteins, while cow's and goat's milk respectively have 32.4 g/l and 30.2 g/l. In this respect the concentration of milk proteins varies according to season, stage of lactation, and number of putdown [31]. The average lactose content of camel's milk is 49.8 g/l as well. However, cow's milk contains 42 g/l lactose and goat's milk contains only 17 g/l of lactose. To sum up, lactose content of camel's milk appears to rely not only on the breed, but also on both the stage of lactation and the state of hydration [31].

Additionally ashes content of analyzed milk is equal to 8.06 g/l. Therefore, it appears to be higher than that of cow's milk 7.6 g/l and higher than that of the goat's milk 7.4 g/l. Consequently, results are seemingly within the range of work recorded by Siboukeur (2007) [14] 7.3g/l. Camel's milk ashes content decreased due to water deprivation according to Yagil (1958) [32]. Further, it also varies depending on the stage of lactation [32].

Camel's milk represents a vitamin C value of 27.53 mg/l. Similarly cow's milk is also rich in vitamin C; it contains a value of 20 mg/l. Farah et al., 1992 [33], reported a similar value 37.4 mg/l of vitamin in dromedary milk, while Mahaia et al., 1994 [18], released significantly lower proportions (24.9mg /l). Despite this variability, it is noticed that the vitamin C content of camel's milk is higher than that of bovine milk which is around 20 mg/l. This characteristic further enhances the nutritional value of dromedary milk.

The results (Table 1) illustrate that camel's and goat's milk have a high content of polyphenols 35.45 mg GAE /l, 39.2 mg GAE /l. Cow's milk contains only 28 mg GAE /l. In this case, this variability in polyphenol contents of the samples analyzed is probably related to the phenolic content of the ingested food. Additionally, the collected camel's milk is rich in flavonoids 29.05 mg EQ/l whereas cow's and goat's milk contain respectively 25.3 mg EQ /l and 31.3 mg EQ/l. The presence of flavonoids in dromedary's milk may also be due to the nature and composition of the plants consumed. Consequently multiple analyses allowed us to conclude that vitamin C, lactose, fats, proteins, and density have high variable values compared to other parameters.

### 3.2. Nutritional Daily Recovery of Camel, Goat, and Cow Milk

Table 2 reveals the daily needs of human insured by camel's milk, goat's milk, and cow's milk. Hence, we can deduce that camel's milk covers the nutritional needs of human in (fats, proteins, lactose, and vitamin C). Accordingly, camel's milk can be considered as rich in nutrients and vitamin necessary for the health of human. Further, camel's milk chemical composition appears to be richer than those of cow's and goat's milk which agree with the work of Sboui (2009) [16], which showed richness in proteins. In this respect the difference may be due to animal feeding, environmental conditions, and lactation stage [34]. Analyzed camel's milk is richer in protein and slightly in fats than that of cows. These components, as described by Guliye (2007) [35], delineate important variations according to the stage of lactation and the species. Besides, camel's milk is richer in lactose (the major milk carbohydrate) than that in cattle, and this has been described in other studies by Abu-lehia (1989) [36]. In line with this, the concentration of lactose varies during the lactation period: it is low at birth (2.8%) but increases during the first 24 hours and reaches 5% as long as the quantity of water required is available [37].

Camel's milk is the essential basis in the diet of nomads, as well as being a cure for many diseases. In addition, the biochemical composition of camel's milk is therefore compared with that of other species, in order to understand some therapeutic uses. As defined, camel's milk is exceptionally rich in vitamin C, niacin, essential amino acids (valine, leucine and phenylalanine), unsaturated fatty acids, antimicrobial factors (lysozyme, lactoperoxidase and lactoferrin), prostaglandins, and insulin. It is used in the treatment of human tuberculosis, diabetes, liver disease, respiratory disorders, child diarrhea, gallstones, nervous disorders, general fatigue, and gastric ulcers. Further several scientific studies have confirmed the therapeutic properties of camel's milk, which is known for its antioxidant richness, which has a protective role against free radicals that have harmful effects on human health.

### 3.3. Comparative Study of the Physicochemical and Biochemical Characteristics of Camel Milk from North Africa and East

A comparative study of physicochemical and biochemical characteristics of camel's milk from North Africa and East has been made. The results shown in Table 3 have been processed by the PCA.

**Figure 2:**
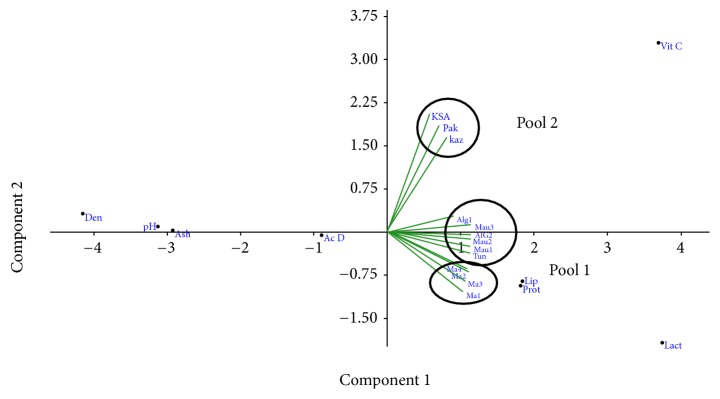
PCA representation of dromedary milk types and their physicochemical and biochemical characteristics represented on two components.

Figure 2 illustrates two main components (component 1 and component 2) that summarize the data in Table 3.

Figure 2 points out that physicochemical characteristics (pH, Den, Ash, and AcD) contribute negatively to component 1, with an order of Den, pH, ashes, and AcD. As regards biochemical analysis, it is inferred that fats (Lip), proteins (Prot), lactose (lactose), and vitamin C take part positively to component 1 for biochemical analysis. Additionally it should be noticed that lactose and vitamin C also contribute to component 2 while vitamin C contributes positively and lactose contributes negatively. Moreover fats and proteins are highly correlated and contribute positively to component 1. As for different types of dromedary milk, it can be deduced that milk from North Africa is highly linked with each other and makes a pool (pool 1) formed by camel milk from Morocco (Ma), Algeria (Alg), Tunisia (Tun), and Mauritania (Mau). Camel milk of Algeria (Alg), Tunisia (Tun), and Mauritania (Mau) compete mainly to component 1. Milk from Morocco contributes positively to component 1 and negatively to component 2. Regarding milk from the orient, Saudi Arabia, Pakistan, and Kazakhstan form a pool (pool 2) which contributes mainly to component 2. Pool 2 is characterized by high levels of vitamin C in contrast with pool 1, which is characterized by elevated levels of fats, proteins, and lactose. Hence these results confirm the regional diversity of Moroccan camel's milk (Table 1). Regional diversity is peculiar to Morocco, wherein there are several climates [38].

Our principal component analysis (PCA) agrees with variance analysis (study of camel milk collected from different regions of Morocco in comparison with goat's and cow's milk), which confirms that camel milk from North Africa differs from that of the East at the physicochemical and biochemical characteristics. To explain this difference, it is well known that milk yields and their nutritive composition are affected by several factors, such as forage quantity and quality, watering frequency, climate, breeding age, parity, milking frequency, calf nursing, milking method (hand or machine milking), health, and reproductive status [39–46]. For instance, according to Musaad, Faye, and Abu Nikhela, 2013 [47], camels that calved during the cold months (November to February) were most productive, with the highest persistency, peak yield, and longest lactation length. Besides, the effect of lactation period on the composition of camel milk has been studied in four dromedaries in Kazakhstan. The fat content varied from 4.34% to 7.81% with a slight decrease all along the lactation and a minimal value at the 14th week corresponding to the lactation peak. Moreover, the variation in protein content was from 2.58% to 3.64% and the minimal protein value has been observed at the 14th week of lactation. Further, lactose varied slightly around its mean of 3.46%. (Konuspayeva et al., 2007) [48]. Other studies have shown that total milk solids, milk fat content, and milk pH depend on milking interval, decreased with an increasing milking interval, profiling the greatest value at 8 h (14.1±0.4%, 4.6±0.5%, and 6.66±0.05, respectively) and the lowest at 24 h intervals (12.3±0.9%, 2.9±0.6%, and 6.54±0.02, respectively). Milk protein (3.9±0.1%), lactose (4.5±0.2%), ash (0.84±0.01%), and density (1.028±0.01) are constant for all milking intervals. Lactose concentration (67±32 *μ*mol) has been constant from 8 to 16 h but increased dramatically at 24 h intervals (338±118 *μ*mol), indicating that mammary tight junctions have become permeable after 24 h of milk accumulation [49]. Data from different studies have displayed that casein N, whey protein N, and nonprotein N ranged from 61 to 76%, 17 to 29%, and 5.8 to 10.6%, respectively. This illustrates that normal camel milk has slightly higher contents of whey proteins and nonprotein N [18, 50, 51]. In the other side, the ash contents in dromedary are similar to those of cow and goat, but lower than in sheep milk. This may be due to the effect of feeding as well as the types of fodder grazed by camels (Yagil, 1985) [32].

Chemical composition in Jordanian camel milk (*Camelus dromedarius*) has been monitored in Jordan over one year; the analysis included total solids, fat, protein, vitamins, minerals, and organic acids. Large seasonal variations in total solids and fat have been apparent with maxima in mid-winter of 139 and 39.0 g/l, respectively, and minima in August of 102 and 25.0 g/l. These differences may be sufficient to alter the sensory properties of the milk. The mean values of trace elements like zinc (5.8 mg/l), iron (4.4 mg/l), and manganese (0.05 mg/l) in Jordanian camel milk could provide valuable additions to the diet of urban populations, as could the mean concentration of vitamin C (33 mg/l). The levels of organic acids have been generally higher than in bovine milk [52].

## 4. Conclusion 

Milk takes an equally important place in the daily eating habits seeing its balanced composition in basic nutrients and its richness in vitamin and minerals. This work exhibits that Moroccan dromedary milk presents a regional diversity in terms of nutrition from the south (Dakhla) to the north (Errachidia and Fes-Meknes). Internationally, Moroccan camel's milk belongs to a group (North African pool) characterized by the richness of macronutrients in terms of fats, proteins, and lactose. Eastern milks (KSA, Pakistan, and Kazakhstan) are characterized by high vitamin C content. Consequently, physicochemical characteristics have no effect on the distribution of the camel's milk analyzed.

## Figures and Tables

**Table 1:** Study of camel milk collected from different regions of Morocco and their comparison with goat's and cow's milk.

Milk	Camel sources				Templates	
Parameters	Dakhla	Fez-Meknes	Errachidia	Average	Cow	Goat
pH	6.43±0.07 ^b^	6.60±0.01 ^a, c^	6.50±0.01^b^	6.51±0.085	6.79±0.01	6.70±0.01
Density (kg/l)	1.028±0.001	1.029±0.0005	1.03±0.0005	1.029±0.001	1.036±0,001	1.031±0.001
Acidity (°D)	15±1	17±1^a^	13±1	15±2	18±1	17±1
proteins (g/l)	32.2±0.750	33±0.115	32.6±0.351	32.6±0.4	32.4±0.529	30.2±0.067
fats (g/l)	39±1 ^a, b^	33±1^c^	34.9±0.550^b, c^	35.6±3.066	36±1	41±1.050
Sugar (lactose) (g/l)	56±1.154 ^a^	45.6±0.503	47.8±0.416^c^	49.8±7.186	42±0.577	17±1
Ashes (g/l)	7.9±0.1	8±0.1	8.3±0,404	8.06±0.321	7.6±0.152	7.4±0.060
Flavonoids (mgEQ/l)	30±0.529 ^b^	27±1^c^	30.15±0.145	29.05±1.776	25.6±0.321	31.3±0.20
Polyphenols (mgGAE/l)	37.85±0.15^b, a^	33±1 ^a, c^	35.5±0.404^c, b^	35.45±2.425	28±1	39.2±0.2
Vitamin C (mg/l)	30±1^a^	29.6±0,351^a^	23±1^c, b^	27.53±3.93	20±1	10.7±0.264

^a^: p<0.05 vs. Errachidia.

^b^: p<0.05 vs. Fez-Meknes.

^c^: p<0.05 vs. Dakhla.

**Table 2:** Daily recovery (fats, proteins, lactose, and vitamin C) insured by milk of the camel and goat's and cow's milk.

Chemical elements	Milk source			Recovery (%)*∗*		
	Camel	Goat	Cow	Camel	Goat	Cow
Fats (g/l)	39	3.02	32.4	144.4	120	120
Proteins (g/l)	32.2	4.1	36	46	5.8	51.4
Lactose (g/l)	56	1.7	42	37.3	1.1	28
Vitamin C (mg/l)	30	10.7	20	3300	1200	2100

*∗*Recovery calculated based on (G Potier de Courcy et al., 2010).

**Table 3:** Physicochemical and biochemical characteristics of camel milk from North Africa and East.

Parameters	Ma1	Ma2	Ma3	Ma4	Alg1	Alg2	Tun	Mau1	Mau2	Mau3	KSA	Pak	Kaz
pH	6.5	6.6	6.43	6.47	5.67	6.65	6.41	6.47	6.38	6.33	6.6	6.77	6.45
Acidity (°D)	13	17	15	19	41.9	16.75	17.2	15.2	14.45	13.25	14	18	26.6
Density (g/l)	1.030	1.029	1.028	1.026	1.026	1.032	1.020	1.027	1.028	1.028	ND	1.015	1.03
fats (g/l)	34.9	33	39	27.2	29.9	28	37.5	32.4	27.4	33.2	32	26.3	59.6
Proteins (g/l)	32.6	33	32.2	25.5	ND	35.68	34.15	33.2	33.1	34.1	29	25.4	34.6
Lactose (g/l)	47.8	45.6	56	43.7	28.2	43.12	42.78	43.2	44.2	36.1	44	36.5	30
Ashes (g/l)	8.3	8	7.9	8.7	ND	7.2	7.5	6.3	6.5	6.1	7.9	7.9	ND
Vitamin C	23	29.9	30	ND	ND	41.4	ND	37.4	39.7	42.1	1170	ND	154

Ma1. Ma2. Ma3: Maroc1. Maroc2. Maroc3 (this work).

Ma4: Maroc 4 (Ismaili et al,. 2016) [13].

Alg1: Algeria1 (Siboukeur, 2007) [14]; Alg2: Algeria2 (Debbouz and al,. 2014) [15].

Tun: tunisia (Sboui et al,. 2009) [16].

Mau1. Mau2. Mau3: Mauritania1. Mauritania2. Mauritania3 (Ould Moustapha Abdellahi and Ould Hamad Sidi, 2016) [17].

K.S.A.: kingdom of Saudi Arabia (Mehaia, 1995) [18].

KAZ: Kazakhstan (Konuspayeva, 2007) [19].

PAK: Pakistan (Khaskheli, 2004) [20].

## Data Availability

The data used to support the findings of this study are included within the article.
